# Blood Thiol Redox State in Chronic Kidney Disease

**DOI:** 10.3390/ijms23052853

**Published:** 2022-03-05

**Authors:** Maria Lisa Garavaglia, Daniela Giustarini, Graziano Colombo, Francesco Reggiani, Silvia Finazzi, Marta Calatroni, Lucia Landoni, Nicola Marcello Portinaro, Aldo Milzani, Salvatore Badalamenti, Ranieri Rossi, Isabella Dalle-Donne

**Affiliations:** 1 Department of Biosciences (Department of Excellence 2018–2022), Università degli Studi di Milano, Via Celoria 26, 20133 Milan, Italy; maria.garavaglia@unimi.it (M.L.G.); graziano.colombo@unimi.it (G.C.); lucia.landoni@studenti.unimi.it (L.L.); aldo.milzani@unimi.it (A.M.); 2 Department of Biotechnology, Chemistry and Pharmacy (Department of Excellence 2018–2022), University of Siena, Via A. Moro 2, 53100 Siena, Italy; daniela.giustarini@unisi.it; 3 Nephrology and Dialysis Unit, IRCCS Humanitas Research Hospital, Via Manzoni 56, Rozzano, 20089 Milan, Italy or francesco.reggiani@hunimed.eu (F.R.); silvia.finazzi@humanitas.it (S.F.); or marta.calatroni@hunimed.eu (M.C.); salvatore.badalamenti@humanitas.it (S.B.); 4 Department of Biomedical Sciences, Humanitas University, Pieve Emanuele, 20090 Milan, Italy; 5 Department of Medical Biotechnologies and Translational Medicine, Università degli Studi di Milano, 20133 Milan, Italy; nicola.portinaro@unimi.it

**Keywords:** chronic kidney disease, end-stage renal disease, haemodialysis, peritoneal dialysis, protein thiols, *S*-thiolated proteins, protein thiolation index

## Abstract

Thiols (sulfhydryl groups) are effective antioxidants that can preserve the correct structure of proteins, and can protect cells and tissues from damage induced by oxidative stress. Abnormal levels of thiols have been measured in the blood of patients with moderate-to-severe chronic kidney disease (CKD) compared to healthy subjects, as well as in end-stage renal disease (ESRD) patients on haemodialysis or peritoneal dialysis. The levels of protein thiols (a measure of the endogenous antioxidant capacity inversely related to protein oxidation) and *S*-thiolated proteins (mixed disulphides of protein thiols and low molecular mass thiols), and the protein thiolation index (the molar ratio of the *S*-thiolated proteins to free protein thiols in plasma) have been investigated in the plasma or red blood cells of CKD and ESRD patients as possible biomarkers of oxidative stress. This type of minimally invasive analysis provides valuable information on the redox status of the less-easily accessible tissues and organs, and of the whole organism. This review provides an overview of reversible modifications in protein thiols in the setting of CKD and renal replacement therapy. The evidence suggests that protein thiols, *S*-thiolated proteins, and the protein thiolation index are promising biomarkers of reversible oxidative stress that could be included in the routine monitoring of CKD and ESRD patients.

## 1. Introduction

Chronic kidney disease (CKD) affects approximately 10% of the adult population worldwide [1]. In 2016, it was the ninth most common cause of death in high-income countries, and epidemiological projections—considering the demographic trends and lifestyles of this population—suggest that the mortality rates due to this pathology will more than double globally by 2040 [2]. Therefore, the development of interventional strategies to support the prevention and management of CKD is of primary importance for the health system. According to the Kidney Disease Improving Global Outcomes [3], CKD is classified into five stages based on the glomerular filtration rate (GFR), which represents the best index of renal function, and three stages based on albuminuria levels. The rationale of this classification is the strong correlation between the GFR and albuminuria values and the prognosis of CKD [3]. CKD—regardless of the underlying aetiology—is slowly progressive, and several factors (i.e., uncontrolled hypertension and diabetes, proteinuria, inflammation, obesity) may accelerate the progression [4]. In a small percentage of patients, the disease can progress to end-stage renal disease (ESRD, CKD stage 5), in which renal replacement therapy (i.e., haemodialysis, peritoneal dialysis, and/or kidney transplantation) is necessary to ensure survival. Haemodialysis (HD), peritoneal dialysis (PD), and kidney transplantation have distinct impacts on patients’ quality of life, and are associated with different clinical outcomes and adverse events [5].

CKD is associated with a higher risk for cardiovascular mortality, as well as all-cause mortality. Many experimental and clinical studies, of which a complete overview was provided in a recent review [6], have shown that the amplification of oxidative stress occupies a central position in the intricate and intertwined thread of the mechanisms involved in the pathogenesis of CKD, and that the disease progression is associated with a significant increase in reactive oxygen species (ROS) generation. It is well established that patients with CKD show a significantly increased oxidative stress compared to healthy subjects, which is present from the early stages of the disease and during its progression, which can result from a series of causal factors that influence and exacerbate each other in a holistic vicious circle [7].

The kidney is a metabolically very active organ, the epithelial cells of which are rich in mitochondria in which redox reactions occur. Furthermore, ROS play an important role in the physiological regulation of renal function. This makes the kidney particularly vulnerable to damage caused by oxidative stress, which can accelerate the progression of kidney disease. Oxidative stress can be induced and enhanced in the context of the uremic milieu [8,9,10,11,12,13,14,15], and of CKD-associated chronic low-grade inflammation [16,17,18,19]. Oxidative stress can be further exacerbated by other pro-oxidant conditions which are frequently present in moderate-to-severe stages of CKD, and are even more in ESRD (e.g., advanced age, diabetes, dyslipidemia, hypertension, obesity, and infectious complications) [20,21,22], and by the lower levels of water-soluble antioxidant vitamins measured in CKD patients due to the dietary restriction of fresh fruits and vegetables required of them in order to avoid hyperkalaemia [23,24]. Furthermore, HD or PD can lead to increased oxidative stress [17,25], which is instead mitigated by restoring renal function after kidney transplantation [26,27].

In the last two decades or so, oxidative stress has been increasingly related to the onset and/or progression of a growing number of human diseases. For this reason, there is a considerable research interest in the identification of oxidative stress biomarkers that can have diagnostic, prognostic, and therapeutic value, for an ever-earlier prediction of the disease onset and/or for the monitoring of disease progression. According to the BEST resource provided by the FDA-NIH Biomarker Working Group, [28], a clinically useful biomarker should be able to be measured reliably and reproducibly (it must be reasonably stable, present in accessible tissues or fluids, and cost-effective). Furthermore, it must show specificity for the disease, have prognostic value, or be correlated with the activity (progression, regression, and outcome after the intervention) of the disease. Regarding biomarkers of oxidative stress, the increase in oxidative damage can be detected by the identification of stable oxidation end products produced by different reaction pathways, because the in vivo detection of circulating ROS is extremely difficult due to their high biological reactivity and short half-life.

Carbonylated proteins, nitrated protein, protein-bound di-tyrosines, advanced oxidation protein products [29,30,31,32,33,34,35], and protein thiols (P-SH) [36] have been investigated as reliable biomarkers of oxidative stress in correlation with diseases. Other oxidative stress biomarkers have been discussed in a recent comprehensive review, to which reference should be made for an exhaustive description that goes beyond the objectives of this text [37].

In the presence of oxidative stress, the nucleophilic functional groups (such as -OH, -NH_2_ and -SH) of proteins, polysaccharides and nucleic acids can be oxidized. Due to its chemical characteristics, -SH is more reactive than -OH and -NH_2_; consequently, it is more prone to oxidation or conjugation. As far as proteins are concerned, P-SH can oxidize to sulfenic acid (P-SOH). The latter can be irreversibly oxidized either to sulphinic (P-SO_2_H) or to sulphonic (P-SO_3_H) acids [29]. Alternatively, P-SOH can be converted back to a disulphide through reactions with low molecular mass thiols (LMM-SH), e.g., homocysteine, cysteinylglycine, cysteine and glutathione, leading to the formation of protein/LMM-SH mixed disulphides, also known as *S*-thiolated proteins. These processes eventually allow the return to the P-SH reduced form [29].

Thiol-based redox systems protect cells and organisms against ROS, maintain redox homeostasis in various cellular compartments, and contribute to redox signalling and redox regulation [38]. Here, it is interesting to note that protein S-thiolation has a dual physiological role: the storage of LMM-thiols and the regulation of protein activity [39,40]. Moreover, *S*-thiolation is considered an adaptive response to protect P-SH from the loss of biological activity due to irreversible oxidation (i.e., further oxidation to P-SO_2_H and P-SO_3_H) [29,39,41]. Although protein *S*-thiolation is considered a defensive mechanism, it can also have pathological consequences in particular conditions. For instance, the proteins *S*-homocysteinylation and protein *S*-glutathionylation are recognized risk factors for vascular diseases [42,43]. 

The levels of P-SH and *S*-thiolated proteins in plasma or in isolated red blood cells (RBCs) have been suggested as biomarkers of oxidative stress in some human diseases, such as chronic obstructive pulmonary disease and asthma [44], alkaptonuria [45], cardiovascular diseases [43], and physiological ageing [46]. Furthermore, over the past two decades, numerous studies have highlighted the change in the levels of several oxidative damage biomarkers, such as plasma P-SH and *S*-thiolated proteins in CKD stages 1–4 (i.e., patients with different degrees of CKD but with residual renal function), and in ESRD patients. Recent reviews summarize the experimental evidence demonstrating the involvement of ROS in the progression of chronic renal failure, the oxidative damage produced at the level of RBCs in chronic renal failure, and the ways in which these factors may be related to the onset of major systemic comorbidities such as cardiovascular disease (CVD) [47,48,49].

The aim of this review is to provide an update of the most relevant observational studies that have investigated P-SH and *S*-thiolated proteins in the plasma and RBCs of CKD patients, and the ways in which their levels change in the progression from moderate to severe stages of CKD, and in patients with ESRD on HD and PD. Because the search for screening markers that enable the early diagnosis and more effective monitoring of CKD is still ongoing, a complete review on this topic could allow us to create a wider vision to overcome the technical and economic difficulties that preclude the use of oxidative stress biomarkers in clinical practice for CKD. Success in this area would allow the identification of individuals who are at risk, as well as the prediction of the short- and long-term results of any therapeutic approach. 

## 2. Protein Thiols and *S*-Thiolated Proteins in Plasma and *RBCs*

Multiple systems are involved in plasma redox signalling. These systems do not have the same sensitivity to oxidants or respond the same way to antioxidants. Therefore, for completeness and clarity, we present here a brief overview of the thiols and *S*-thiolated proteins present in plasma and RBCs, before addressing the review of the known alterations of P-SH and *S*-thiolated proteins in patients with CKD.

**Figure 1:**
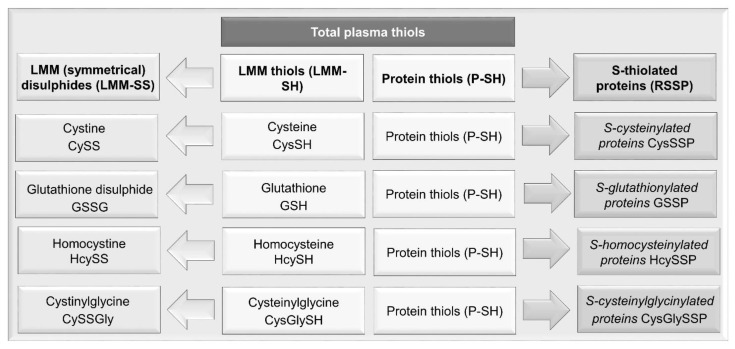
Nomenclature of thiols. The nomenclature of the different redox forms of the main thiols in human plasma. The sum of P-SH and LMM-SH constitutes the total pool of plasma thiols. *S*-thiolated proteins: RSSP, where R indicates a generic low molecular mass thiol (LMM-SH, e.g., homocysteine, cysteinylglycine, cysteine or GSH.

**Figure 2:**
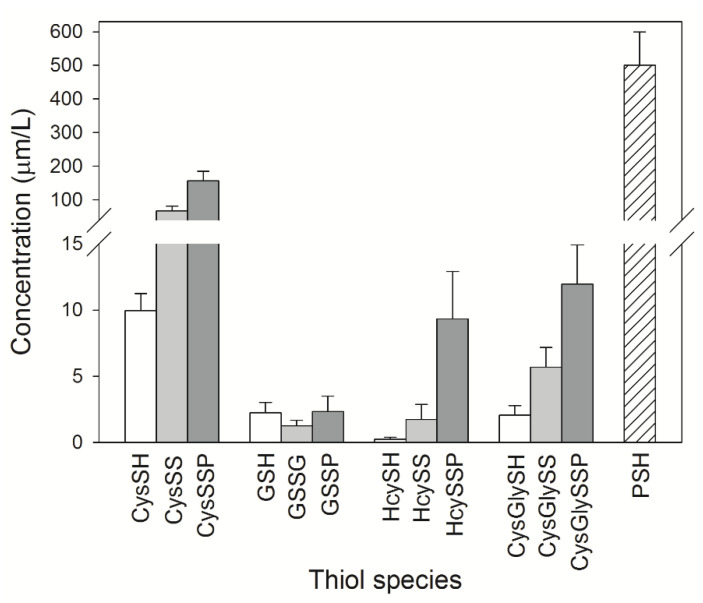
Plasma thiol levels. The concentrations of LMM-SH (white bars), LMM-SS (grey bars), *S*-thiolated proteins (dark grey bars) and P-SH in healthy subjects, based on the data in the references [41,50,64,70].

Plasma thiols can be categorized into P-SH and LMM-SH which are not bound to protein; the latter can generate both low molecular mass symmetrical disulphides (LMM-SS) and mixed disulphides with proteins (or *S*-thiolated proteins, RSSP) (Figure 1). LMM-SH—i.e., homocysteine, cysteinylglycine, cysteine and glutathione (GSH)—are present in the plasma of healthy subjects at a low concentration range of 0.1–12 μM, and all together constitute only 12–20 μM [41,50]. The major thiol/disulphide redox pool in mammalian plasma is the cysteine/cystine couple. Free cysteine (CysSH) has a physiological plasma concentration of approximately 10 µM [50]. It is susceptible to oxidation, forming disulphides with itself (cystine, CySS) or with protein thiols. As Cys-SH is more reactive than GSH in thiol-disulphide exchange with plasma proteins [51], the latter are mainly reversibly *S*-cysteinylated (Figure 2). 

The plasma levels of total cysteinylglycine (CysGlySH + its oxidised forms) and total homocysteine (HcySH + its oxidised forms) are higher than those of GSH, the main intracellular antioxidant and comprised, respectively, between 18–36 μM and 5–15 μM [52]. Hyperhomocysteinemia is an important risk factor for vascular disease and atherosclerosis [6,53,54,55,56,57], as well as for autoimmune diseases [58] and neurological disorders [59]. Higher plasma concentrations of total homocysteine are caused by both genetic and non-genetic factors: among the latter, we can mention renal failure [60].

Recently, in subjects without CKD (*n* = 4745) who participated in the Prevention of End-stage REnal and Vascular Disease (PREVEND) study, elevated serum free thiol concentrations—adjusted to the total serum protein—were shown to be associated with a lower risk of the new-onset of CKD in this population, constituting a possible marker for the identification of the individuals who are most at risk of developing the disease. Therefore, the assessment of the serum free sulfhydryl status would be useful for preventive purposes, in order to mitigate the future burden of CKD in the general population [61].

HcySH can bind reversibly to P-SH, forming *S*-homocysteinylated proteins (HcySSP). Moreover, HcySH can also be converted to its cyclic thioester, forming the Hcy-thiolactone, which reacts with the epsilon-amino group of Lys residues in protein, thus generating *N*-homocysteinylated protein [62]. It has been proposed that the *N*-homocysteinylation of low-density lipoproteins (LDL) can contribute to an acceleration of the atherogenic process in subjects with hyperhomocysteinaemia [63]. Most of the GSH in human blood is found inside RBCs (approximately 3 mM per RBC), as its plasma concentration is in the µM range, and leukocytes—which contain 2–4 mM GSH—are much less numerous than RBCs. In healthy erythrocytes, 98% of glutathione is present in its active reduced form, GSH [64], and protects RBC proteins from irreversible oxidation by maintaining –SH groups of haemoglobin in the reduced state (Hb-SH) [65]. 

The GSH/GSSG redox potential is lower in the erythrocytes of haemodialyzed patients than in those of healthy controls [66]. In addition, serum from haemodialyzed patients has decreased GSH levels [67] and a diminished activity of the antioxidant enzymes superoxide dismutase and GSH peroxidase [68]. Under conditions of moderate or severe oxidative stress, erythrocytic proteins (e.g., haemoglobin or membrane skeletal proteins) may be prevalently *S*-glutathionylated in a reversible manner [36,69].

In the plasma of healthy adults, the concentration of P-SH ranges from 400 to 600 μM [41,70], and is mainly due to the Cys34 residue of albumin, which—due to its high plasma concentration (about 600 μM)—accounts for 80% of plasma –SH groups [71]. The plasma albumin pool consists of a mixture of mercaptalbumin, bearing the free thiol and non-mercaptalbumin, bearing the modified thiol. Considering that, in healthy adults, about 70% of total albumin is mercaptoalbumin, its –SH group reaches concentrations higher than 400 μM. Thus, the albumin Cys34 thiol is an important scavenger of ROS in the vascular compartment, and therefore a quantitatively important redox buffer of human blood [41,71,72,73,74,75], acting as an effective extracellular antioxidant because of its multiple ligand binding capabilities [72]. These properties are closely related to the structure and redox state of the molecule [76]. The non-mercaptoalbumin pool consists mainly of mixed disulphides of albumin with LMM-SH [41]. In the presence of moderate or severe oxidative stress, the reduced albumin (Alb-SH) concentration decreases, and that of *S*-cysteinylated albumin increases, whereas a low level, or absence, of oxidative stress induces the opposite changes, i.e., a higher level of Alb-SH and a lower level of *S*-cysteinylated albumin.

## 3. P-SH and *S*-Thiolated Proteins in the Moderate-to-Severe Stages of CKD

Several studies have examined the plasma P-SH levels in patients with moderate-to-severe stages of CKD compared with healthy subjects, by spectrophotometry (Table 1). A pioneering study by Himmelfarb and colleagues showed that the plasma P-SH levels were significantly lower in the CKD patient group, likely because of oxidative stress [77].

Successively, lower plasma P-SH levels in CKD patients were correlated to lower plasma albumin levels (hypoalbuminaemia) [78], suggesting a causal relationship between hypoalbuminaemia and lower plasma P-SH levels. A positive association between P-SH and the estimated glomerular filtration rate (eGFR), and a negative correlation between P-SH and creatinine was also demonstrated in adult CKD patients in a recent study, which also detected a significant reduction in P-SH in stage 5 CKD patients [87]. However, the albumin levels were not evaluated, and the control group subjects were significantly younger than CKD patients [87]. 

The levels of total plasma thiols (P-SH + LMM-SH) were measured in a group of patients with CKD stages 1–5, most of whom were stage 3 (46%) and stage 4 (24%): the levels of plasma thiols, adjusted to the plasma albumin concentrations, decreased dependently on the CKD stage [83]. Moreover, a positive correlation between the eGFR and thiol concentration was observed [83]. A decrease in Alb-SH accompanied by a decrease in renal function was confirmed in non-diabetic CKD patients [88]. Decreased thiol and albumin levels have also been reported in a more recent study conducted on a small group of children with stage 3–5 CKD [89]. This study included and grouped data from both haemodialyzed and non-haemodialyzed patients, making it difficult to understand the real burden of the results differentiated for the two groups. However, a positive correlation between the albumin concentration and levels of thiols, (mixed and symmetrical) disulphides, and total thiols was found in the plasma of CKD children. When the thiol, disulphide, and total thiol levels were normalized to the albumin concentration, only the levels of total thiols and thiols were significantly lower in CKD children than in age-matched control subjects. In the CKD group, the levels of thiols, total thiols, and disulphides correlated positively with the albumin concentration, and negatively with the urea concentration, whereas the thiol and total thiol levels were positively correlated with the eGFR and negatively with the creatinine level [89]. Two other recent studies on adult CKD patients not on HD determined that total plasma thiol, and the (“native”) thiol levels were significantly lower in CKD patients than in healthy controls [90,91]. One study also found a significant reduction in the level of disulphides but no significant difference in the disulphide/thiol, disulphide/total thiol, and thiol/total thiol ratios between the CKD and healthy control groups [91]. The other study found a significant variation in disulphide/thiol and disulphide/total thiol ratios only [90]. This discrepancy could be explained by the fact that the control group in the latter study had a significantly higher BMI than the CKD group, causing an increase in oxidative stress in the control group with increased levels of disulphides [92].

Terawaki and colleagues first adopted the redox state of albumin Cys34 measured by an HPLC method as a marker of oxidative stress in pre-dialysis patients with CKD. Their findings indicated that both *S*-cysteinylated albumin and irreversibly oxidized albumin (Alb-SO_2_H and Alb-SO_3_H) increased as renal function, measured as creatinine clearance, decreased [84]. The authors confirmed their findings in a subsequent study, where they also showed that albumin-SH levels progressively decreased with decreasing kidney function [85]. Conversely, the levels of *S*-thiolated albumin increased progressively as kidney function decreased, suggesting that the redox state of albumin progressively changes with the worsening of kidney function. Moreover, they found a significant positive correlation between *S*-thiolated albumin and blood urea nitrogen, and between *S*-thiolated albumin and serum creatinine. Another recent study analysed the serum fraction of oxidized albumin normalized to total albumin (i.e., oxidized and native albumin) in pre-dialysis CKD patients [93]. The correlation between the oxidized serum fraction of albumin, f(HNA), and numerous clinical parameters related to CKD was examined. The study revealed a positive correlation between f(HNA), kidney function and urea nitrogen in the blood, and also with the degree of anaemia of the CKD patients, independent of age; f(HNA) was also significantly and independently associated with the plasma levels of haemoglobin and ferritin. A negative correlation was demonstrated between the levels of xanthine oxidase—an isoform of xanthine oxidoreductase which is thought to increase the cardiovascular burden among CKD patients via oxidative radical production—and the plasma fraction of Alb-SH and reversibly oxidized albumin [94]. These findings suggest that the analysis of the redox state of albumin Cys34 thiol can be a potentially useful method for the assessment of oxidative stress and the degree of kidney dysfunction in CKD patients, although some analytical and instrumental limitations must be overcome.

Elevated levels of plasma HcySH are commonly present in patients with CKD. In a recent study involving 62 patients in different stages of CKD, a negative correlation was found between the plasma HcySH and eGFR in patients with CKD stages III to V, although a significant increase in HcySH levels was present only in subjects with stage V compared to patients with stage II CKD. The plasma Cys/Hcy ratio turned out to be a more sensitive indicator because it was already significantly decreased at stage III of CKD [95]. Zinellu and colleagues reported that HcySH and CysSH bound to LDL were significantly increased in CKD patients, and the plasma levels of *S*-homocysteinylated LDL and creatinine were positively correlated with each other [86]. Based on these results, the authors suggested that the increase in *S*-homocysteinylated LDL levels might account, at least in part, for the excess of cardiovascular risk in CKD patients: *S*-homocysteinylated LDL can therefore be considered a key risk marker of cardiovascular disease (CVD) in CKD patients [86]. Another study detected a significant increase in *S*-cysteinylated and *S*-homocysteinylated plasma proteins in CKD patients compared to healthy subjects [96]. More recently, Zinellu and colleagues evaluated the plasma levels of P-SH and LMM-SH in a group of hypertensive CKD patients at stages 3–4, more than 60% of whom were hyperhomocysteinemic (HcySH > 15 μmol/L), reporting that the thickness of the carotid intima-media was inversely related to plasma levels of P-SH [82].

## 4. P-SH and *S*-Thiolated Proteins in HD Patients Compared to Healthy Subjects

Several studies have examined the plasma levels of P-SH and *S*-thiolated proteins in ESRD patients on maintenance HD compared to healthy subjects; others detected the concentration of *S*-thiolated proteins in the RBCs of HD patients (Table 2).

Himmelfarb and colleagues reported a significant decrease in plasma P-SH in HD patients [77], in line with what has also been observed in other studies (Table 2). For example, the plasma P-SH levels (measured in μmol/L) decreased significantly in hypoalbuminemic HD patients, both compared to healthy subjects and to normoalbuminemic HD patients, as well as between the latter and healthy subjects [97]. The levels of inflammation and oxidative stress biomarkers increased in both groups of HD patients compared to healthy subjects, and further selective increases in levels of inflammation and oxidative stress biomarkers occurred in hypoalbuminemic patients, suggesting that hypoalbuminemia, acute-phase inflammation, and oxidative stress could act synergistically to increase cardiovascular morbidity and mortality risk in HD patients [97]. Significantly lower plasma P-SH levels in hypoalbuminemic HD patients could be due, at least in part, to hypoalbuminemia.

Plasma P-SH has also been investigated in HD patients not receiving iron or recombinant human erythropoietin [99]. Iron deficiency is common in HD patients [106]: intravenous iron administration is one of the cornerstones of anaemia’s treatment, but it could further aggravate oxidative stress [107]. The plasma P-SH levels were found to be lower in HD patients not receiving intravenous iron or recombinant human erythropoietin vs. healthy controls [99]; however, this study did not specify whether the HD patients were normoalbuminemic or hypoalbuminemic.

Koca and colleagues divided HD patients into four groups according to their HD duration (dialysis vintage) [100], reporting that the total plasma thiols were significantly lower in all of the groups of HD patients than in healthy subjects, but no significant difference was found between the groups of HD patients with different dialysis vintages [100].

**Figure 3:**
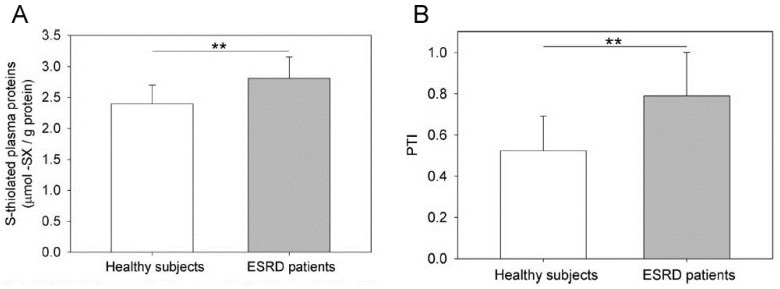
*S*-thiolated plasma proteins and PTI in ESRD patients compared to healthy subjects. (**A**) The plasma proteins of ESRD patients (*n* = 20) and age-matched healthy subjects (*n* = 20) were analysed for their total *S*-thiolated protein content by colorimetric reaction with ninhydrin. The data are expressed as the mean ± SD. The differences between the means of the two groups were evaluated using Student’s *t*-test (** = *p* < 0.001). (**B**) The plasma of both ESRD patients (*n* = 20) and age-matched healthy subjects (*n* = 20) was analysed for PTI. The data are expressed as the mean ± SD. The differences between means of the two groups were evaluated using Student’s *t*-test (** = *p* < 0.001). Reprinted with permission from Colombo et al. [101].

The protein thiolation index (PTI, a new biomarker of oxidative stress that is defined as the molar ratio of the sum of all LMM-SH bound to *S*-thiolated plasma proteins to free P-SH [46,108]) is higher in HD patients than in controls, whereas the concentration of P-SH and that of total plasma thiols are lower in HD patients than in healthy subjects [90,101,105] (Figure 3). P-SH and PTI provide different but complementary information. A low P-SH concentration indicates an absolute loss of protein reducing power but does not provide information on concomitant changes in disulphide forms, while a high PTI indicates a relative excess of oxidized vs. reduced proteins in patients; however, being a ratio, it offers no indication about the absolute amounts of disulphide and reduced forms.

Several studies examined in HD patients the levels of plasma *S*-thiolated proteins [102,103,104,105] and *S*-thiolated haemoglobin in RBCs [66] (Table 2). The plasma levels of Alb-SH and *S*-cysteinylated albumin were determined in HD patients subjected to intravenous iron administration by the HPLC method [84]. HD patients had increased levels of *S*-cysteinylated albumin and irreversibly oxidized albumin (Alb-SO_2_H and Alb-SO_3_H), and decreased levels of Alb-SH [102]. On the other hand, the plasma levels of *S*-homocysteinylated proteins were significantly higher in HD patients than in healthy subjects [104]. This finding could have functional consequences; indeed, the site of interaction with diazepam in homocysteinylated albumin is altered due to the presence of the sulphur amino acid, and the interaction between the drug and the protein is reduced, partly explaining why the albumin-binding capacity is reduced in uraemia [104]. Other investigations determined that ESRD patients show increased levels of HcySH, which have been related to carotid artery atherosclerosis and increased rates of cardiovascular events [109,110]. Other studies have shown that *S*-glutathionylated haemoglobin (HbSSG) was 46% higher in HD patients than in healthy subjects, and that *S*-cysteinylated Hb (HbSSCy) was >3-fold higher in HD patients than in controls [66]. In addition, both HbSSCy and HbSSG were positively correlated with blood urea nitrogen, creatinine, and normalized protein catabolic rate, which are parameters related to both uraemia and nutrition [66].

## 5. P-SH and *S*-Thiolated Proteins in HD Patients before and after an HD Session

Several studies have measured the levels of P-SH and *S*-thiolated proteins in HD patients before (pre-HD) and after an HD session (post-HD) (Table 3). Some authors reported that the levels of total plasma thiols (P-SH + LMM-SH) significantly decreased post-HD, returning to the range measured in healthy subjects [111,112,113]. The plasma P-SH levels in ESRD patients on HD were significantly lower than those in healthy subjects. However, the HD session was associated with an increase in the plasma P-SH levels, such that after HD the concentration of P-SH did not differ from that of healthy subjects. Similar results were also obtained when plasma P-SH were expressed as nanomoles per milligram of protein, calculated using total plasma protein concentrations [112]. This result was also confirmed when considering possible dialysis-induced changes in the plasma protein concentration [113], which can dramatically affect the measured values of total plasma thiols [105]. Moreover, the total plasma thiol levels decreased significantly even after haemodiafiltration [113].

A study reported that the concentration of *S*-cysteinylated proteins in pre-HD ESRD patients was higher than that in controls, and did not change after HD [115]. This result contrasts with that of other studies showing a decrease in *S*-thiolated protein levels post-HD. The concentration of Alb-SH and *S*-cysteinylated albumin were measured in ESRD non-diabetic patients pre-HD and post-HD, compared to healthy subjects, by direct infusion electrospray mass spectrometry [116]. The total amount of albumin isolated from ESRD patients pre-HD was characterized by a significant decrease in the Alb-SH fraction, and by a concomitant increase in the S-cysteinylated albumin fraction; HD significantly decreased the S-cysteinylated albumin and restored Alb-SH in all ESRD patients [116]. A study of 20 patients under dialytic treatment measured 59 ± 3% and 42 ± 2% of *S*-thiolated albumin before and after dialysis, while 39 ± 3% *S*-thiolated albumin was found in healthy subjects [117].

Both the albumin thiol content and the conformational status of albumin were evaluated in CKD patients before and after HD, and were compared with those measured in healthy subjects [81]. The results were consistent with those already observed previously, with a reduction in the albumin thiol level, which was further significantly reduced after HD. The susceptibility to oxidation of plasma albumin isolated from controls and CKD patients before and after HD was assessed by paramagnetic resonance electron spectroscopy as a marker of the conformational modification of the molecule. The results show that albumin in CKD patients is highly modified in vivo, and is not vulnerable to oxidation in the same way as normal albumin, suggesting a possible conformational variation in HD subjects.

**Figure 4:**
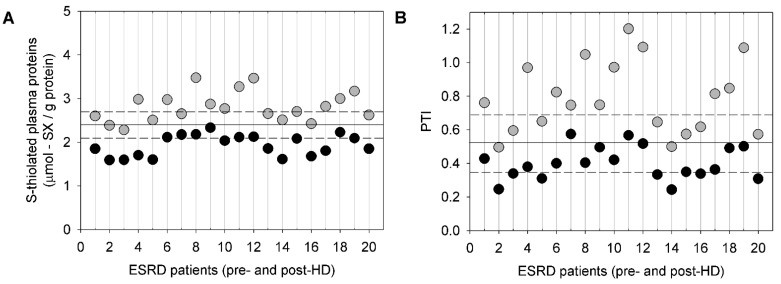
*S*-thiolated plasma proteins and PTI in ESRD patients pre-HD and post-HD. (**A**) The effects of HD on the total plasma thiol levels before (grey circles) and immediately after (black circles) HD were examined in patients with ESRD who received maintenance dialysis (*n* = 20). The horizontal solid line represents the mean of the total plasma thiol level in age-matched healthy subjects. The horizontal dashed lines represent the SD of the total plasma thiol level in age-matched healthy subjects. (**B**) The effects of HD on the *S*-thiolated plasma protein levels before (grey circles) and immediately after (black circles) HD were examined in patients with ESRD on maintenance dialysis (*n* = 20). The horizontal solid line represents the mean of the *S*-thiolated plasma protein levels in age-matched healthy subjects. The horizontal dashed lines represent the SD of the plasma *S*-thiolated plasma protein level in age-matched healthy subjects. Reprinted with permission from Colombo et al. [101].

The total plasma thiols, plasma *S*-thiolated proteins, and PTI were measured in ESRD patients, before and after a regular HD session, and were compared to age-matched healthy subjects [101]. Colombo and colleagues found a significant decrease in the level of total plasma thiols and, conversely, a significant increase in the level of *S*-thiolated proteins in ESRD patients. In most patients, the level of post-HD total plasma thiols did not differ from those of healthy subjects, whereas the plasma level of *S*-thiolated proteins was lower in HD patients than in age-matched healthy controls (Figure 4), suggesting that a single HD session restores the thiol redox status of plasma and the antioxidant capacity of plasma thiols. In addition, HD patients had a significantly higher PTI than the age-matched healthy subjects, and HD was associated with a decrease in PTI to normal, or lower than normal, levels (Figure 4).

## 6. P-SH and *S*-Thiolated Proteins in PD

Only a few studies examined the levels of P-SH and *S*-thiolated proteins in PD. Himmelfarb and colleagues reported that the levels of total plasma thiols in PD patients were significantly different from those measured in healthy subjects (215 ± 19 μM vs. 279 ± 12, *p* = 0.011, *n* = 9 and 10, respectively) but not from those measured in HD patients [77]. Similar results were obtained in a subsequent study [118].

One study reported that continuous ambulatory PD restores the normal level of total GSH and total Cys concentration, whereas it decreases the level of *S*-thiolated proteins (except that of homocysteinylated proteins) in plasma [96]. 

## 7. Conclusions and Perspectives

All of the articles examined in this review provide evidence of a generalized thiol/oxidative stress in patients with CKD, which is reflected in the alterations of the levels of P-SH and *S*-thiolated proteins compared to healthy control subjects. Analyses of the levels of P-SH and *S*-thiolated proteins suggest that oxidative stress is present even in the early stages of CKD; it is augmented in parallel with the progression of the disease to ESRD, and is further exacerbated in HD patients. Oxidative stress is involved in the pathogenesis of CKD progression and complications, particularly cardiovascular diseases. Thus, it represents a non-traditional risk factor for all causes of mortality in patients with CKD and ESRD. For this reason, oxidative stress must become an important diagnostic and prognostic factor, and a target for CKD prevention and treatment [119]. Unfortunately, oxidative stress assessment is still not standardized, and this fact represents an obstacle for its use in everyday clinical practice.

Some studies have shown that the levels of total plasma thiols, measured with Ellman’s DTNB method, diminish in moderate-severe CKD (Table 1) in ESRD patients on HD (Table 2 and Table 3) or on PD. However, the spectrophotometric analysis of the total plasma thiols with Ellman’s method mainly measures P-SH, as they represent the most abundant thiol pool in human plasma, being in the 400–600 μM range, whereas LMM-SH altogether constitute only 12–20 μM [41,50,70]. Therefore, the contribution of LMM-SH to the amount of plasma thiols measured by Ellman’s method with DTNB can be considered negligible. Hence, these findings suggest a decrease in the plasma P-SH levels in patients with CKD, and in ESRD patients on HD or PD (Table 1, Table 2 and Table 3).

Unfortunately, the values of the plasma P-SH levels in CKD (Table 1) and ESRD patients on renal replacement therapy (Table 2 and Table 3), as well as in healthy control subjects, are often shown in different units, e.g., μmol/L, μg/L, and μmol/g protein, making it very difficult to compare the results of various studies conducted in different laboratories. Furthermore, in some studies, the CKD and ESRD patients (Table 1, Table 2 and Table 3) were hypoalbuminemic (with serum albumin levels < 3.8 g/dL), making it particularly difficult to compare P-SH levels, even if they are shown in the same units, between CKD patients and healthy normoalbuminemic subjects. In order to compare the levels of plasma P-SH between CKD patients or ESRD patients on renal replacement therapy and healthy subjects, it is very important to normalize the P-SH concentrations against the total plasma protein or plasma albumin concentrations, e.g., to measure the concentration of the plasma P-SH as μmol -SH/g plasma proteins [44,83,101,114,118]. In this way, the P-SH values would become easily comparable between different studies and, within each individual study, between CKD patients and healthy controls.

Unfortunately, there is no gold standard method for the measurement of the albumin concentration in plasma. Albumin is generally measured using a colorimetric method based on bromocresol-green (BCG) or bromocresol-purple (BCP). Other approaches, e.g., immunonephelometry (INP) or immunoturbidimetry, are used less frequently, as they are more time-consuming and expensive. In addition, there are significant discrepancies when measuring the albumin concentration by different methods (BCG, BCP and INP), especially when determining it in ESRD patients on HD [120]. The albumin concentration measured by BCG is consistently 4–10 g/L higher than that determined by BCP or INP.

Another important point concerning the normalization of the P-SH concentration in relation to the plasma albumin concentration should be mentioned here. In fact, in CKD, albumin can undergo several irreversible post-translational modifications, such as carbonylation [32,121,122] and carbamylation [123,124,125]. Due to the increased urea and isocyanate concentrations in ESRD patients, protein carbamylation (i.e., the covalent binding of isocyanate to the ε-amino group of lysine) increases significantly [126]. For HD patients, the carbamylation of albumin is the main cause of the discrepancy found for values measured by BCP; this leads to an underestimation of 0–6 g/L. Several researchers have proposed that INP, or immunoturbidimetry, would be the preferred method for the measurement albumin in HD patients [120,127,128]. Some have also suggested that the choice of method for the measurement of the plasma albumin concentration (BCP, BCG, INP) could have an important impact on the classification of CKD patients [127].

Based on the results presented in this article, we can conclude that CKD and ESRD patients on HD or PD show lower plasma thiol levels than healthy controls, suggesting that the uremic condition per se plays a key role in oxidative/thiol stress (Table 3). *S*-cysteinylated Cys34 accounts for most of the oxidized forms of albumin in CKD [84,85,101] (Table 1) and in ESRD patients on renal replacement therapy [102,103] (Table 2 and Table 3). CKD patients are among those with the highest risk for CVD [129,130]. CKD and ESRD patients have a 5- to 10-fold higher risk of developing CVD than age-matched healthy controls [131,132]. The Cys34 thiol of albumin represents by far the largest fraction of all plasma thiols; thus, it can have a protective effect on the vascular endothelium, as was already stated above. In this regard, some researchers have assessed whether changes in the redox state of the Cys34 thiol of albumin influence the incidence of CVD in HD patients [133,134]. These authors reported that a lower concentration of Alb-SH was indicative of an increased CV mortality, demonstrating that the redox state of the Cys34 thiol is closely related to CVD in both HD [133,134] and PD patients [135]. The Alb-SH levels were significantly higher in PD patients with high peritoneal membrane transport properties than in those with low peritoneal membrane transport properties [136].

All of these studies suggest that, together with albumin carbamylation [124,125], the redox state of albumin Cys34 thiol is related to the overall risk factor for CVD among ESRD patients. However, care should be taken when interpreting the outcome of these small-scale studies to define the association of the redox status of albumin Cys34 thiol with CVD, as further large-scale studies are required.

Regarding *S*-thiolated plasma protein levels in pre- and post-HD ESRD patients (Table 3), following the HD session, the plasma P-SH levels transiently increase because of the de-thiolation of *S*-thiolated plasma proteins, as there is a decrease in oxidative stress during the HD session. This is very important in the case of albumin, the Cys34-SH of which represents the dominant antioxidant in plasma. However, in the period between two consecutive HD sessions, oxidative plasma conditions induce P-SH to bind LMM-SH, determining the reversible formation of *S*-thiolated plasma proteins. Therefore, *S*-thiolated plasma proteins represent a useful marker of thiol-specific reversible oxidative stress in ESRD patients on HD. Therefore, HD has only a limited and transient beneficial effect on the redox state of plasma P-SH, i.e., HD patients are exposed to periodical peaks of severe oxidative stress.

Individual HD sessions have different effects on different types of biomarkers of protein oxidation. For example, the post-HD levels of plasma protein carbonyls are significantly higher than the pre-HD levels, suggesting an increase in oxidative stress during the HD session [32,137,138,139]. The apparent discrepancy between the low levels of *S*-thiolated proteins (and, consequently, high plasma P-SH levels) post-HD and the high plasma levels of protein carbonyls post-HD is probably because, while protein *S*-thiolation is a reversible process, protein carbonylation is, generally, irreversible [29].

In CKD, RBCs are exposed to oxidative stress and uremic toxins released into the blood. This is particularly evident in ESRD patients, where Hb has been found to be *S*-glutathionylated (Table 2 and Table 3). This fact has clinical relevance because the exposure to oxidative stress and uremic toxins may have a role in the development of anaemia in patients with CKD. In fact, it has been widely demonstrated that the RBCs of CKD/ESRD patients have a reduced lifespan, which is caused by a hastened eryptosis, or RBC rupture, compared to healthy subjects. It was observed that, when blood from normal donors was transfused into uremic patients, reductions in the RBC lifespan occurred [140]. Several studies examined RBCs’ oxidative stress in renal failure. HbSSG has been suggested to be a clinical marker of oxidative stress in ESRD patients undergoing HD [66,141,142] or PD [142]. Moreover, the structure of Hb in ESRD patients is impaired, and these alterations are enhanced during HD [143]. Regarding RBC thiol stress in HD and PD, LMM-SH (i.e., GSH) in the RBCs of patients on continuous ambulatory PD remain at a physiological level [144], whereas the GSH level in RBCs decreases before HD, and increases after HD [144,145]. However, in HD patients the increase in LMM-SH level is short-lasting and limited to a brief post-HD period, while the increase in the level of LMM-SH in the RBCs of PD patients is continuous and persistent [144]. Thus, both PD and HD therapies increase the antioxidant power of RBC, but PD does so continuously, while the effect of HD is short-lasting and intermittent. These results suggest that, as far as thiol stress is concerned, toxic compounds do not accumulate in PD patients, thanks to the continuous dialytic procedure and the fact that PD patients more often maintain a residual diuresis, lowering the severity of oxidative stress [144].

In conclusion, this review provides evidence indicating a decrease in the plasma P-SH levels and an increase in *S*-thiolated plasma proteins and *S*-thiolated RBC proteins in patients with CKD (Table 1, Table 2 and Table 3). The levels of P-SH and S-thiolated proteins, and the PTI are promising biomarkers of reversible thiol/oxidative stress in moderate-to-severe CKD, and in HD and PD. The successful transposition of these biomarkers into a clinical test requires the availability of a simple, artefact-free assay that should be selective, specific, and sensitive. The measurement of these biomarkers is complicated by the complexity of the methodologies that must be applied in order to avoid procedural artifacts, and that require special care in sample handling and preparation. Nevertheless, several methods are available that minimize artifacts and reproducibly measure the amounts of P-SH, *S*-thiolated proteins, PTI, and LMM-SH in cells, tissues, and body fluids [146,147,148,149,150,151]. These promising biomarkers, which are serious candidates for the evaluation of oxidative stress in CKD patients, deserve further exploration in large and appropriately designed prospective studies. When this will be done, the levels of blood P-SH, *S*-thiolated proteins, LMM-SH, and the PTI could be included in the routine monitoring of CKD patients. This will determine important clinical implications, because—at the moment—an assessment of oxidative stress in patients with CKD is not feasible in everyday clinical practice, in spite of its relevance in CKD progression and complications development.

## Figures and Tables

**Table 1:** P-SH and *S*-thiolated proteins in the plasma or serum of patients with moderate-to-severe stages of CKD. All of the data are expressed as means ± SD, unless otherwise indicated. (a) Median, with interquartile range in parentheses. The data of *S*-thiolated proteins are in grey cells. *** *p* < 0.001, ** *p* < 0.01, * *p* < 0.05.

Concentration		Albumin Concentration mg/dL		Additional Information	Reference
CKD Patients	Control Group	CKD Patients	Control Group		
202 ± 20 μM (*n* = 10)	279 ± 12 μM (*n* = 10)	-	-	CKD patients not receiving renal replacement therapy	[77]
182.3 ± 15.9 μg/L (*n* = 24) ***	286.4 ± 21.8 μg/L (*n* = 20)	-	-	albumin significantly lower in CKD patients compared to healthy controls (*p* < 0.0001) Analyses performed in serum	[78]
304.0 ± 55.2 μM (*n* = 184) ***	328.4 ± 33.3 μM (*n* = 43)	4.3 ± 0.38	4.4 ± 0.22	CKD patients at stages 3, 4	[79]
191.26 ± 16.75 μM (*n* = 41) **	342.34 ± 43.43 μM (*n* = 41)	2.1 ± 1.0	4.4 ± 1.3	CKD patients on conservative treatment Analyses performed in serum	[80]
6.3 ± 0.9 μmol/g protein (*n* = 16) *	7.3 ± 0.8 μmol/g protein (*n* = 13)	-	-	Non dialysis CKD patients	[81]
3.59 (3.31–4.80) μmol/g protein (a) (*n* = 24)	-	-	-	CKD hypertensive patients stages 3, 4	[82]
stage 2: 9.8 ± 3.5 nmol/mg albumin stage 3: 9.9 ± 2.6 nmol/mg albumin stages 5: 7.9 ± 2.5 nmol/mg albumin (*n* = 68)	-	-	-	-	[83]
69.44 ± 7.26% (*n* = 55)	-	4.1 ± 0.4	-	Predialysis patients with CKD	[84]
28.59 ± 6.93%				S-thiolated albumin	
stages 1, 2 77.2 ± 3.4% (*n* = 7) stage 3a 75.5 ± 3.8% (*n* = 7) stage 3b 71.5 ± 3.3% (*n* = 6) stages 4, 5 66.2 ± 4.1% (*n* = 12)	-	stages 1, 2 4.0 ± 0.1 stage 3a 4.0 ± 0.1 stage 3b 4.1 ± 0.3 stages 4, 5 4.1 ± 0.3		-	[85]
stages 1, 2 21.0 ± 3.4% (*n* = 7) stage 3a 22.4 ± 4.1% (*n* = 7) stage 3b 26.2 ± 3.1% (*n* = 6) stages 4, 5 31.1 ± 4.1% (*n* = 12)				S-thiolated albumin	
633 ± 248 nM	430 ± 153 nM			S-cysteinylated + S-homocysteinylated LDL Analyses carried out by capillary electrophoresis laser-induced fluorescence detection	[86]

**Table 2:** P-SH and S-thiolated proteins in the plasma or serum of ESRD patients on HD. All of the data are expressed as means ± SD, unless otherwise indicated. Data regarding *S*-thiolated proteins are in the grey cells. (b) Median with interquartile range in parentheses; (c) median with the 25–75th percentile in parentheses. HbSSCy, cysteinylated haemoglobin; HbSSG, *S*-glutathionylated haemoglobin; PTI, protein thiolation index.

Concentration		Albumin Concentration g/dL		Additional Information	Reference
HD Patients	Control Group	HD Patients	Control Group		
178 ± 18 μM (*n* = 10) ***	279 ± 12 μM (*n* = 10)	-	-	-	[77]
normoalbuminemic patients 299.28 ± 8.64 μM (*n* = 18) *** hypoalbuminemic patients 229.00 ± 9.43 μM (*n* = 18) ***	428.39 ± 8.61 μM (*n* = 18)	normoalbuminemic patients 4.2 ± 0.02 hypoalbuminemic patients 2.9 ± 0.03	-	-	[97]
174.9 ± 10.5 μg/L (*n* = 32) ***	286.4 ± 21.8 μg/L (*n* = 20)	albumin significantly lower in CKD patients compared to healthy controls (*p* < 0.0001)	-	Dialysis vintage 16 ± 4 months Analyses performed in serum	[78]
280 ± 11 μM (*n* = 28)	416 ± 6 μM (*n* = 49)	3.78 ± 0.6	-	-	[98]
170.79 ± 12.31 μM (*n* = 22) ***	286.4 ± 21.8 μM (*n* = 20)	-	-	Dialysis vintage 16 ± 4 months Analyses performed in serum	[99]
164.12 ± 13.54 μM (*n* = 48) **	342.34 ± 43.43 μM (*n* = 41)	2.8 ± 0.2	4.4 ± 1.3	Dialysis vintage 16 ± 4 months Analyses performed in serum	[80]
Figure 3 (*n* = §) ***	Figure 3 (*n* = 24)	-	-	-	[100]
3.72 ± 0.66 μmol/g protein (*n* = 20) ***	4.73 ± 0.71 μmol/g protein (*n* = 20)	7.37 ± 0.49 (a)	7.61 ± 0.386	Dialysis vintage 4.4 ± 1.9 months	[101]
36.0 ± 6.03% (*n* = 22)	64.6 ± 0.4% (*n* = 11)	-	-	Dialysis vintage 1–9 years	[102]
29.7 ± 0.5%	29.7 ± 0.5%			S-cysteinylated albumin	
40.4 ± 8.7% (*n* = 20) **	53.6 ± 6.4% (*n* = 10)	-	-	Dialysis vintage 1–10 years	[103]
49.7 ± 8.0%) **	38.7 ± 6.3%	-	-	S-cysteinylated albumin	
825.53 ± 121.08 *** pmol/mg (*n* = 28)	139.52 ± 15.43 pmol/mg (*n* = 14)	-	-	Dialysis vintage > 6 months S-homocysteinylated proteins	[104]
mean 0.76 (min–max 0.61–0.88) (b) (*n* = 71) ***	mean0.43 (min–max 0.40–0.54) (b) (*n* = 24)	3.49 ± 0.38	4.23 ± 0.31	PTI	[105]
19.3 ± 4.80 pmol/mg Hb (*n* = 33) ***	13.2 ± 2.79 pmol/mg (*n* = 21)			HbSSG	[66]
11.5 (9.6–17.2) pmol/mg Hb (c) ***	38.3 (29.0–63.3) pmol/mg Hb (c)			HbSSCy	[66]

*** *p* ≤ 0.001; ** *p* ≤ 0.01. (a) Total plasma protein. § The patients were divided into four groups according to the HD duration: group 1, 0–2 years of treatment (*n* = 31); group 2, 3–5 years of treatment (*n* = 40); group 3, 6–8 years of treatment (*n* = 27); group 4, 9–11 years of treatment (*n* = 13). There was no significant difference in the mean age between the groups. The plasma –SH levels were significantly lower in all of the patient groups than in controls (all groups *p* < 0.001).

**Table 3:** P-SH and S-thiolated proteins in the plasma of HD patients before and after HD. All of the data are expressed as means ± SD, unless otherwise indicated. The data regarding *S*-thiolated proteins are in grey cells. (b) Median with interquartile range in parentheses. Pre-HD, before HD; post-HD, after HD; PTI, protein thiolation index; M, male; F, female.

Concentration		Albumin Concentration g/dL		Additional Information	Reference
HD Patients	Control Group	HD Patients	Control Group		
pre-HD 268 ± 22 μM post-HD 425 ± 15 μM (*n* = 11)	438 ± 16 μM (*n* = 17)	-	-	Polysulfone membrane Dialysis vintage 49 ± 11 months	[112]
pre-HD 265 ± 19 μM post-HD 408 ± 23 μM (*n* = 11)	438 ± 16 μM (*n* = 17)	-	-	Cellulose triacetate membrane Dialysis vintage 49 ± 11 months	[112]
pre-HD 312.4 ± 60.5 μM post-HD 435.6 ± 78.9 μM (*n* = 55) ***	451.1 ± 90.1 μM (*n* = 25)	7.1 ± 0.4 g/L (a)	6.9 ± 0.6 (a)	Dialysis vintage 86.6 ± 75.1 months	[111]
pre-HD 339 (54) μM (b) post-HD 493 (94) μM (b) (*n* = 19)	512 (58) μM (a) (*n* = 17)	-	-	standard bicarbonate hemodialysis	[113]
pre-HDF 343 (48) μM (b) post-HDF 529 (110) μM (b) (*n* = 25)	512 (58) μM (a) (*n* = 17)	-	-	hemodiafiltration	[113]
pre-HD 9.56 ± 2.5 mol/mg protein post-HD 11.17 ± 2.2 mol/mg protein (*n* = 10) *	8.59 ± 0.7 mol/mg protein (*n* = 9)	-	-	-	[114]
pre-HD 254.62 ± 62.1 μM post-HD 258.32 ± 68.2 μM (*n* = 21)	213.21 ± 42.0 μM (*n* = 15)	-	-	S-cysteinylated albumin Dialysis vintage 1–9 years	[115]
pre-HD 58 ± 7% post-HD 37 ± 7% (*n* = 8) **	27 ± 4% (*n* = 10)	3.77 ± 0.36	4.12 ± 0.57	S-thiolated albumin Dialysis vintage 38.6 ± 71.8 months	[116]
pre-HD 0.79 ± 0.21 after-HD from 23.0 to 61.5% decrease (mean 48.17 ± 9.17) (*n* = 20)	0.52 ± 0.17 (*n* = 20)	pre-HD 7.37 ± 0.49 (a) post-HD 7.89 ± 0.72 (a)	7.61 ± 0.386 (a)	PTI Dialysis vintage 4.4 ± 19 months	[101]

*** *p* ≤ 0.001; ** *p* ≤ 0.01; * *p* ≤ 0.05. (a) Total plasma protein.

## Abbreviations

Alb-SH	reduced albumin
CKD	chronic kidney disease
CVD	cardiovascular disease
CysGlySH	cysteinylglycine
CySS	cystine
DTNB	5,5-dithiobis(2-nitrobenzoic acid)
eGFR	estimated glomerular filtration rate
ESRD	end-stage renal disease
GSH	glutathione
HbSSCy	cysteinylated haemoglobin
HbSSG	glutathionylated haemoglobin
HcySH	homocysteine
HD	haemodialysis
LDL	low-density lipoprotein
LMM-SH	low molecular mass thiols
PD	peritoneal dialysis
PSH	protein thiols
PTI	protein thiolation index
RBCs	red blood cells
ROS	reactive oxygen species
